# Patterns of Medication Regimens and Drug-Drug Interactions Among Older Adults Requiring Long-Term Care

**DOI:** 10.1093/geroni/igaf122.4170

**Published:** 2025-12-31

**Authors:** Shotaro Hagiwara, Jun Komiyama, Masao Iwagami, Shota Hamada, Hiroyuki Kobayashi, Nanako Tamiya

**Affiliations:** University of Tsukuba, Mito, Ibaraki, Japan; University of Tsukuba, Tsukuba, Ibaraki, Japan; University of Tsukuba, Tsukuba, Ibaraki, Japan; Tokyo University of Pharmacology and Life Sciences, Hachioji, Tokyo, Japan; University of Tsukuba, Mito, Ibaraki, Japan; University of Tsukuba, Tsukuba, Ibaraki, Japan

## Abstract

**Purpose:**

Although older patients requiring long-term care (LTC) are highly susceptible to adverse drug events, they often experience polypharmacy, leading to potential drug-drug interactions (DDIs). This study examined the patterns of medication regimens and the risks of DDIs among older patients requiring LTC.

**Methods:**

We analyzed the medication regimens among 67,531 individuals aged 65 and older who utilized long-term care insurance services in Ibaraki Prefecture, Japan, based on public medical and LTC insurance claims data in 2018. We examined DDIs using package inserts and DrugBank Online (version 5.1.13). An association analysis was conducted using R (version 4.3.3) to calculate Support, Confidence, and Lift values. Medication classification followed the WHO’s ATC classification.

**Results:**

The median number of drugs was seven (IQR, 5-9). The most frequent two-drug combination was gastric acid-related disorder drugs and calcium channel blockers (CCBs) (Support 0.33, Confidence 0.73, Lift 1.02). For four-drug combinations, it was antithrombotic drugs, CCBs, renin-angiotensin system agents, and gastric acid-related disorder drugs (0.07, 0.78, 1.09). For six-drug combinations, it was antithrombotic drugs, CCBs, renin-angiotensin system agents, lipid-modifying drugs, psychotropic drugs, and gastric acid-related disorder drugs (0.01, 0.84, 1.17). Among the top 30 drugs, co-prescribed medications with Support>0.03 were extracted. The likelihood of being prescribed at least one drug with a moderate or major interaction was highest for clopidogrel (90.1%), zolpidem (88.1%), rosuvastatin (85.9%), celecoxib (84.2%), and spironolactone (83.4%).

**Conclusion:**

We identified the most common medication regimens among older patients requiring LTC and high-risk drugs for DDIs. Establishing a prescription check system is needed.

